# Effect of prednisolone on live birth rate in women with unexplained recurrent pregnancy loss: a study protocol for a double-blind, placebo-controlled, multicentre, randomised controlled trial (PREMI-study)

**DOI:** 10.1136/bmjopen-2024-096545

**Published:** 2025-06-19

**Authors:** Yentl Béquet, Marie-Louise van der Hoorn, Michael Eikmans, Renate Van der Molen, Saskia le Cessie, Nan van Geloven, Elske van den Akker-van Marle, Marloes Vermeulen, Merel van den Berg, Jan-Peter de Bruin, Astrid Cantineau, Dana Huppelschoten, Tess Meuleman, Annemarie Mulders, Salwan Al-Nasiry, Gijs Teklenburg, Harold Verhoeve, Jantien Visser, Moniek van der Zanden, Mariëtte Goddijn, Eileen Lashley

**Affiliations:** 1 Department of Gynecology and Obstetrics, Leiden University Medical Center, Leiden, Zuid-Holland, The Netherlands; 2 Laboratory of Reproductive Immunology, Department of Immunohematology and Blood transfusion, Leiden University Medical Center, Leiden, Zuid-Holland, The Netherlands; 3 Department of Laboratory Medicine, Laboratory for Medical Immunology, Radboud University Medical Center, Nijmegen, Gelderland, The Netherlands; 4 Department of Clinical Epidemiology, Leiden University Medical Center, Leiden, Zuid-Holland, The Netherlands; 5 Department of Biomedical Data Sciences, Leiden University Medical Center, Leiden, The Netherlands; 6 Patient association "Freya”, Asperen, The Netherlands; 7 Centre for Reproductive Medicine, Department of Gynecology and Obstetrics, Amsterdam Reproduction and Development Research Institute, Amsterdam UMC Location AMC Department of Obstetrics Gynecology, Amsterdam, Noord-Holland, The Netherlands; 8 Department of Gynecology and Obstetrics, Jeroen Bosch Hospital, ’s-Hertogenbosch, Noord-Brabant, The Netherlands; 9 Department of Gynecology and Obstetrics, University Medical Center Groningen, Groningen, The Netherlands; 10 Department of Gynecology and Obstetrics, Catharina Hospital, Eindhoven, Noord-Brabant, The Netherlands; 11 Department of Gynecology and Obstetrics, Radboud University Medical Centre, Nijmegen, The Netherlands; 12 Department of Gynecology and Obstetrics, Erasmus Medical Center, Rotterdam, Zuid-Holland, The Netherlands; 13 Department of Gynecology and Obstetrics, Maastricht UMC+, Maastricht, The Netherlands; 14 Department of Gynecology and Obstetrics, Isala Hospital, Zwolle, Overijssel, The Netherlands; 15 Department of Gynecology and Obstetrics, OLVG, Amsterdam, Noord-Holland, The Netherlands; 16 Department of Gynecology and Obstetrics, Amphia Hospital, Breda, The Netherlands; 17 Department of Gynecology and Obstetrics, Medisch Centrum Haaglanden, Den Haag, Zuid-Holland, The Netherlands

**Keywords:** Clinical Trial, Drug Therapy, GYNAECOLOGY, Reproductive medicine, IMMUNOLOGY, Pregnant Women

## Abstract

**Introduction:**

Recurrent pregnancy loss (RPL) is defined as the occurrence of two or more spontaneous pregnancy losses from the time of conception until 24 weeks of gestation. Currently, an underlying cause can be identified in only a minority of the losses. Potentially, an impaired maternal immune response targeting the semiallograft pregnancy may lead to miscarriage. While prior studies have explored the use of immune-suppressing corticosteroids to modulate the maternal immune system and hopefully improve pregnancy outcome, the absence of sufficiently powered randomised controlled trials (RCT) underscores the need for further research. The primary aim of this study is to evaluate if prednisolone administration in early pregnancy (20 mg daily for 6 weeks, then tapering doses for 2 weeks) in women with unexplained RPL leads to a higher live birth rate (LBR) in comparison to placebo. Additionally, the study assesses the tolerability, safety and the cost-effectiveness of this intervention. Finally, we will explore the effect of prednisolone in various subgroups (based on maternal age, number of previous pregnancy losses, presence of specific antibodies and pre-pregnancy endometrial immune cell level).

**Methods and analysis:**

This ongoing multicentre, double-blind RCT will randomise 490 women with unexplained RPL and pregnancy <7 weeks to receive either prednisolone or placebo. Each participant will be followed up for 1 year, with digital questionnaires to assess depression, anxiety, medical expenses and productivity loss. We will also collect data on maternal and paternal demographics and neonatal outcomes. The sample size of 490 participants was calculated according to a minimally important increase in LBR of 12% (expecting a LBR of 63% in the general RPL population), including loss to follow-up (estimated at 5%). The analysis will follow the intention-to-treat principle.

**Ethics and dissemination:**

This study was submitted under the Clinical Trial Regulation (CTR) in Clinical Trials Information System (CTIS) for assessment by the Central Committee on Research Involving Human Subjects (CCMO) under Clinical Trial number: 2023-503220-76-01. It received full approval on 29/01/2024. Study findings will be presented at conferences and published in a peer-reviewed journal. Participants will be informed about the results by publishing them on the publicly available website of the study.

**Trial registration number:**

This trial is registered in ClinicalTrials.gov (ID NCT05725512) and in CTIS (2023-503220-76-01).

**STRENGTHS AND LIMITATIONS OF THIS STUDY**

- This is the first adequately powered randomised controlled trial to investigate the effect of prednisolone on live birth rate in women with unexplained RPL.
- In addition, we study the tolerability, safety and the cost-effectiveness of this intervention, to promote implementation of study results in daily practice.
- The study allows to explore the effect of prednisolone amongst various decidual immune cell populations.
- A limitation of the study might be that the study is underpowered to detect differences in effects between subgroups. Further research must then be conducted with targeted designs and adequate sample sizes to specifically investigate and validate the observed effect within relevant subgroups.

## Introduction

 The most common complication in pregnancy is miscarriage, defined as the spontaneous loss of the foetus before 24 weeks of gestation.^1^ The overall chance for pregnancy loss is between 10% and 15% if maternal age is <35 years.^2^ Recurrent miscarriage, also known as recurrent pregnancy loss (RPL), is defined as two or more (non-)consecutive losses before 24 weeks of gestation. RPL affects approximately 3% of couples trying to conceive.^3^

Underlying factors contributing to RPL include a variety of conditions: maternal antiphospholipid syndrome, congenital uterine abnormalities, parental balanced translocations, thyroid antibodies and endocrine abnormalities (eg, diabetes).^4^,^13^ Additionally, numerous risk factors are associated with RPL, including advanced maternal age,^14^ abnormal body mass index (BMI; either high or low),^15 16^ smoking and other intoxications^17 18^ and specific medication usage.^18^,^20^ These risk factors contribute to abnormal fetal chromosomal development (eg, triploidy and autosomal trisomy^21^). Indeed, 50% of sporadic pregnancy losses are caused by fetal aneuploidy. In women with a higher number of pregnancy losses, however, the likelihood of a euploid pregnancy loss rises with each subsequent pregnancy loss.^22^ Presently, routine genetic analysis of miscarriage tissue is not recommended, leaving approximately 50% of recurrent miscarriages without an underlying factor. Limited understanding of the mechanisms underlying RPL leads to a lack of effective interventions. Numerous studies have thoroughly explored treatments for couples facing unexplained RPL, including human chorionic gonadotropin therapy,^23^ acetylsalicylic acid,^24^ heparin,^24 25^ progesterone^26^ (which is now only indicated in the case of vaginal blood loss in pregnancy after three or more pregnancy losses^27^) and vitamin supplementation.^28^ Immune-related interventions, namely paternal leucocyte immunisation^29^ and intravenous immunoglobulin,^30^ also showed no improvement in the LBR. Consequently, current clinical management is primarily focused on providing supportive care.^3^

As the foetus is a semiallograft, which is accepted by the maternal immune system in healthy pregnancy, many studies proposed the involvement of immunological mechanisms in RPL. These include the level and function of uterine natural killer (uNK) cells, macrophages and regulatory T cells.^31^,^34^ Glucocorticoids, such as prednisolone, could potentially improve the intrauterine environment and implantation by positively influencing these cells to adopt a more tolerogenic phenotype towards the embryo. Glucocorticoids therefore represent a potentially useful therapy in patients with RPL with suspected underlying immunological cause. So far, three randomised controlled trials (RCT) studied the effect of prednisolone on pregnancy outcome in patients with RPL, showing hopeful results.^35^,^37^ However, these trials were inadequately powered to investigate effectiveness, their inclusion criteria differed and in two trials, heparin and acetylsalicylic acid were used as co-intervention. In addition, most patients were selected based on the NK cell density in prior uterine biopsy, though this has not yet proven to be a valid biomarker.^38^ Considering the potential risks and consequences associated with administering glucocorticoids,^39^ a clinical trial within the general population suffering from unexplained RPL with adequate randomisation and power is urgently needed.

### Objective

To examine whether administering prednisolone before 7 weeks of gestation to women with unexplained RPL results in higher live birth rate (LBR) compared with the administration of placebo treatment.

## Methods and analysis

### Design and setting

The PREMI-study is an ongoing—the study started on 29-01-2024 (actual), primary completion 29-07-2026 (estimated) –multicentre, double-blind, placebo-controlled RCT that is being conducted in 12 academic and non-academic centres within the Netherlands.

### Participants and eligibility criteria

Eligible patients are women aged 18–39 years with ‘unexplained recurrent pregnancy loss’, defined as the loss of two or more pregnancies, without a known cause for RPL (such as antiphospholipid syndrome, congenital uterine abnormalities or abnormal parental karyotype). As fetal aneuploidy is the most common cause of pregnancy loss, but more often not tested in case of pregnancy loss, women with potentially aneuploid losses are eligible to participate. The diagnosis of unexplained is based on the latest ESHRE guideline (including biochemical pregnancy losses).^3^ Patients who meet any of the following criteria will be excluded from participation in this study:

- Instable, or exacerbation of, auto-immune diseases such as thyroid disease or inflammatory bowel disease.
- Diabetes mellitus requiring medical treatment.
- Current treatment with systemic corticosteroids (for any indication) or other immunosuppressive medication.
- Simultaneous enrollment in any other trial that studies the effectiveness of an intervention in RPL or previous enrolment in the PREMI trial.
- Contraindications to prednisolone use.
- Use of drugs with known interaction with prednisolone.

Patients can enroll in the study either during early pregnancy (<7 weeks gestation), with randomization performed at enrollment, or before conception if they ave an active desire to conceive, with randomization delayed until pregnancy is confirmed. Both women who conceive spontaneously and those who conceive through artificial reproductive techniques are eligible for participation.

### Procedures, recruitment and randomisation

The total (planned) enrolment period of the study is 30 months, with a subsequent 12 month follow-up. Eligible patients will be identified by local research coordinators and/or staff members trained in ‘good clinical practice’ at participating hospitals. Patients will receive verbal, written and/or video-animated information. When signing the informed consent form, non-pregnant patients will be requested to collect and send in menstrual blood from the first 24 hours of menstruation to the coordinating centre (Leiden University Medical Centre, LUMC) for the isolation of endometrial immune cells. Upon confirmation of pregnancy (urine or blood test) <7 weeks gestational age, randomisation takes place, in a 1:1 ratio, using Castor EDC with variable randomisation blocks (4, 6 and 8). Patients will be assigned to the intervention or placebo group, with allocation blinded to investigators, participants, clinicians and research coordinators.

Data will be collected in a web-based registration system, Castor (CDMS 2023.4). Once a patient is randomised, the research nurse will collect data in the electronic case report form provided in Castor. Database cleaning will be conducted by internal consistency checks and identification of database entries outside expected ranges. Patients will receive standard care with their own treating physician or midwife. All collected data will be coded, processed and stored with adequate precautions to ensure patient confidentiality. This is described in a separate data management plan.

### Intervention and placebo

The intervention entails an 8-week prednisolone or placebo treatment regimen, administered orally in the form of tablets (manufactured by the clinical pharmacist of the Leiden University Medical centre), starting before 7 weeks of gestational age. This treatment includes a daily dosage of 20 mg for 6 weeks, followed by 1 week at 10 mg daily, and concluding with 1 week at 5 mg daily. The placebo and prednisolone treatments are identical in appearance: white circular flat-faced tablets, with a break line, an average weight of 160 mg and a diameter of 8 mm. The prednisolone tablets and placebo tablets are identical in pharmaceutical form and qualitative composition (except for the active substance prednisolone). The tablets contain either 20 mg of prednisolone for the first 6 weeks or 5 mg tablets for the tapering phase. Upon receiving their medication, participants will be given comprehensive instructions to ensure proper usage and adherence to the treatment regimen. Patients are considered compliant when at least 80% of the prescribed treatment regimen is followed (monitored in the medication diary).

### Baseline information

The baseline information is collected at randomisation in Castor EDC and it encompasses:

- Maternal medical history and demographics: number of previous pregnancy losses, age, parity, BMI and relevant medical conditions.
- Paternal information and demographics: biometric data, history of subfertility or infertility, and relevant intoxications.

### Outcome measures

The following outcomes and variables will be collected during this trial;

- Primary outcome: live birth, defined as the birth of a living child beyond 24 weeks of gestational age.
- Pregnancy course monitoring: ultrasound biometric outcomes, gestational age at time of pregnancy loss (if applicable).
- Pregnancy complications, such as hypertensive disorders and gestational diabetes.
- Delivery and postpartum details, including delivery method and postpartum complications.
- Neonatal information: weight, percentile, presence of congenital abnormalities, gestational age and other relevant factors.

Additionally, digital questionnaires will be administered to patients at various intervals during the follow-up period. These include:

- Hospital Anxiety Depression scale and EQ-5D-5L at randomization, 3, 6, and 12 months post-randomization to assess maternal quality of life.
- iMedical Consumption Questionnaire at 6 and 12 months post-randomization to evaluate maternal medical expenses.
- iProductivity Cost Questionnaire (iPCQ) at 6 and 12 months to measure maternal productivity loss.
- Medication diary: common side effects of prednisolone (nausea and vomiting, mood swings, hypertension, hyperglycaemia and weight gain) will be monitored using digital medication diaries to evaluate compliance and adverse reactions.

#### Monitoring and safety

The study is a Consortium study supported by the Dutch Consortium for Healthcare Evaluation and Research in Obstetrics and Gynaecology (NVOG). It receives support and assistance from the Trial Office (Zorgevaluatie Nederland) and has established a Data and Safety Monitoring Board (DSMB) in recognition of the study’s moderate risk level.

This independent DSMB will perform ongoing safety surveillance, a planned safety evaluation (after 100 randomisations) and monitoring of serious adverse events (SAEs). The DSMB’s recommendations will be communicated solely to the study sponsor.

Prednisolone, a corticosteroid with anti-inflammatory and immunosuppressant qualities, is a commonly administered drug. Much experience has been gained with this drug, also in pregnancy, for treating rheumatoid arthritis, asthma or bowel disease.^40^ In addition, some women are given this medication during pregnancy as part of a transplantation regimen. Thus, side effects are well established and known. In pregnancy, prednisolone is metabolised in the placenta to the inactive prednisone. Only 10% of active compound reaches the fetus.^41^ When the use is chronic and in high dosages (>10 mg daily), it is possibly associated with fetal growth restriction and in the third trimester with neonatal adrenal cortical insufficiency.^42^ Based on available studies and two registries, there is substantial evidence showing that prednisolone in the dosage used in the study (20 mg) and usage in the first trimester is safe for mother and fetus. Animal studies showed an association with cleft palate when high dosages of dexamethasone were used; earlier studies have not shown an increased risk for congenital abnormalities with prednisolone in humans.^43 44^ The most common side effects comprise mood changes, weight gain, indigestion, hypertension and hyperglycaemia. In an earlier feasibility trial, none of the side effects were severe enough for women to stop taking medication, and no suspected unexpected serious adverse reactions (SUSAR) or adverse fetal outcomes were reported.^35^

### Sample size calculations

For this superiority trial, we based the sample size on an absolute treatment effect of 12%; in between the by patients and healthcare practitioners predefined, minimally important difference of 10% and a maximal expected difference of 18%. The latter number is based on the treatment effect observed in the meta-analysis by Dan *et al*.^45^ The validity of this meta-analysis is, however, questionable,^46^ and the effect was shown only in a subpopulation of patients with high uNK cell density. We therefore decided on a more conservative approach and calculated the sample size based on a treatment effect of 12%. To demonstrate this increase in the LBR (χ^2^ test, power 80%, two-sided α 0.05) from 63% in the control group (as observed in an earlier large trial in Dutch population^26^) to 75% in the prednisolone group, we need to include 464 patients. Assuming a 5% loss to follow-up, the total number of participants required is 490, with 245 in each arm.

### Statistical analysis

#### Data analysis

We engaged the expertise of statisticians and methodologists to ensure meticulous planning of the data analysis. Additionally, a health technology assessment (HTA) expert was employed to contribute to the cost-effectiveness analysis (CEA) of the research.

The analysis of the gathered data will involve several methodological approaches:

- Demographic information about the studied population will be presented using descriptive statistics, depicted in tables.

The primary outcome is live birth. We will first report the observed number (and percentage) of live births in the treatment and placebo groups. Following the recommendations of the The United States Food and Drug Administration (FDA) for covariate adjustment in clinical trials,^47^ to increase power, we compared the LBRs between the intervention and control group using a multivariable logistic regression analysis, taking into account strong predictors of pregnancy loss, in this case, maternal age and previous number of pregnancy losses.^3^ From this multivariable model, we will derive an estimate of the marginal treatment effect, expressed as an absolute risk difference, along with 95% CI, using standardisation and bootstrapping. The statistical significance threshold will be set at p<0.05, and all analyses will be performed using SPSS or R.

Secondary outcomes and subgroup analysis

Next to the primary outcome, we will consider the following secondary outcomes

- Pregnancy loss, defined as the spontaneous demise before the 24th week of gestation.
- Ongoing pregnancy, defined as viability of the fetus beyond the 12th week of gestation.
- Tolerability (defined as percentage of non-compliance due to side-effects).
- Safety of prednisolone (including congenital abnormalities, gestational weight, SAEs and SUSARs).
- Side-effects to the mother or the fetus and the cost-effectiveness of the treatment.

Again, following the recommendations of the FDA for covariate adjustment in clinical trials,^47^ to increase power, we compared the secondary outcomes between the intervention and control group using a multivariable logistic regression analysis, taking into account strong predictors of pregnancy loss (maternal age and previous number of pregnancy losses).^3^ From this multivariable model, we will derive an estimate of the marginal treatment effect, expressed as an absolute risk difference, along with 95% CI, using standardisation and bootstrapping. The statistical significance threshold will be set at p<0.05.

In addition, we will perform subgroup analyses to explore the effect of prednisolone in the following participant categories:

- Women with positive thyroid peroxidase antibodies.
- Women with positive antinuclear antibodies.
- Women with high and low levels of prepregnancy endometrial uNK cells, macrophages and regulatory T cells*.*
- Participants age (≤36 or >36 years old).
- Number of previous pregnancy losses (≤3 or >3).

We will employ appropriate statistical tests to assess secondary outcomes such as safety parameters, side-effects and quality of life measurements. Depending on the nature of the data (continuous or categorical), t-tests, χ^2^ tests or regression analyses will be applied.

A detailed statistical analysis plan, developed with the expertise of the employed statisticians, will be published prior to the closure of the database on www.zorgevaluatienederland.nl/premi (publicly available).

### Economic evaluations

The CEA will involve an evaluation of the economic impact of prednisolone vs placebo administration. Both a CEA and a cost-utility analysis (CUA) will be performed from a societal and healthcare perspective. This analysis takes into account both the costs incurred and the health outcomes achieved, using established economic evaluation methods.

### Patient and public involvement

Current research question is recommended by the European Society of Human Reproduction and Embryology^3^ to study in further research. In our Dutch ‘NVOG research agenda’ 2023–2026, it is ranked in the Top 12 of most urgent knowledge omissions. The Dutch patient organisation for couples with fertility problems, Freya, is involved in defining this research agenda. Therefore, we collaborate closely with Freya on the development and execution of the PREMI study. This includes proofreading of the research proposal and patient information and help on a prospective preference assessment, to measure potential participants’ stated willingness and identify and evaluate substantial deterrents to participation. During the execution phase, Freya will publish study information on their (social) media and provide feedback for relevant questions or posts. Finally, Freya is involved in the organisation of a patient day on RPL.

## Trials management and monitoring

### Ethics and dissemination

The full protocol of this RCT was reviewed by the Central Committee on research Involving Human Subjects (CCMO) under the Clinical Trial Regulation (CTR) and given approval on 29/01/2024 (CTIS 2023-503220-76-01). Additionally, it received local approval of the Ethical Committee and the board of the LUMC on 17/10/2023 (L23-030).

A manuscript with results of the primary objective of this RCT will be published in relevant peer-reviewed journal. Separate publications will follow on the results of subgroup analyses (immune cell levels) and the economic evaluation.
