# Supplementary material for: Effect of prednisolone on live birth rate in women with unexplained recurrent pregnancy loss: a study protocol for a double-blind, placebo-controlled, multicentre, randomised controlled trial (PREMI-study)
Source: BMJ Open. 2025 Jun 19;15(6):e096545. doi: 10.1136/bmjopen-2024-096545 (PMC12182118; doi:10.1136/bmjopen-2024-096545)
Supplement: Supplementary file 1 [file bmjopen-2024-096545.reviewer_comments.pdf]

PEER REVIEW HISTORY

BMJ Open publishes all reviews undertaken for accepted manuscripts. Reviewers are asked to complete a checklist review form and are provided with free text boxes to elaborate on their assessment. These free text comments are reproduced below.

ARTICLE DETAILS

Title (Provisional)

Effect of prednisolone on live birth rate in women with unexplained recurrent pregnancy loss; a study protocol for a double-blind, placebo-controlled, multicenter, randomized controlled trial (PREMI-study)

Authors

Béquet, Yentl; van der Hoorn, Marie-Louise; Eikmans, Michael; Van der Molen, Renate; le Cessie, S; van Geloven, Nan; van den Akker-van Marle, Elske M; Vermeulen, M.; van den Berg, MMJ; de Bruin, J.P.; Cantineau, Astrid EP; Huppelschoten , Dana; Meuleman, Tess; Mulders, Annemarie; Al-Nasiry, Salwan; Teklenburg, G; Verhoeve, H.R.; Visser, J.; van der Zanden, M.; Goddijn, Mariëtte; Lashley, Eileen

---

VERSION 1 - REVIEW

**Reviewer**	**1**
**Name**	**SUN, XIAOXI**
**Affiliation**	**Shanghai JIAI Genetics and IVF Institute**
**Date**	**08-Jan-2025**
**COI**	**None**

1.For patients with recurrent pregnancy loss, if the previous miscarriage was caused by fetal aneuploidy, would such patients be included?

2.Is biochemical pregnancy loss considered recurrent miscarriage?

3.A research status paragraph should be added in the paper.

**Reviewer**	**2**
**Name**	**Wang, Rui**
**Affiliation**	**The University of Sydney, NHMRC Clinical Trials Centre**
**Date**	**19-Feb-2025**
**COI**	**None**

This is overall a clearly written protocol. The authors may wish to consider the following comments.

1. Timing of randomisation. According to page 7 Line 35-38, there are two possible timepoints for randomisation. “Patients may enrol in the study either during early pregnancy (<7 weeks of gestation) or before conception if they have an active desire to conceive. Both women who conceive spontaneously and those who conceive through artificial reproductive techniques are eligible for participation”. This will create a problem for the timing of randomisation, as those enrolled before conception would have been enrolled much earlier. In addition, for those enrolled before conception scheduled for ART, the time until conception will be uncertain as it may take multiple cycles before a conception occurs.

2. Duration of intervention and follow-up. Following #1, the duration of intervention and follow-up could be much longer for those who are enrolled before conception.

3. Stratified randomisation. If the authors were to keep the inclusion of those enrolled before conception, a stratified randomisation considering the timing or randomisation is recommended and this should also be considered in the analysis.

4. Terminology. Miscarriage and pregnancy loss are interchangeably used across the protocol. It would be better to use “pregnancy loss” consistently across the protocol, in line with the ESHRE guideline on RPL.

5. Outcomes:

- There are some inconsistencies in the outcomes listed in the protocol and those in the trial registration, please make them consistent.

- “gestational age at miscarriage (if applicable)” is defined as an outcome in the protocol. What about those with a live birth? How will this outcome be handled in the analysis?

6. Statistical analysis:

- Any plans for per protocol or as-treated analysis? If so, how was “compliance” defined in this placebo-controlled trial?

- For secondary outcomes, please make it clear whether logistic regression adjusting for prognostic factors will be used for binary outcomes (most secondary outcomes) and whether estimate of the marginal treatment effect (absolute risk difference) will be presented.

- Page 11, line 29-37. These refer to outcome definitions instead of statistical analysis and therefore should be merged to the “outcome measures” section.

7. Subgroup analysis: Are thyroid Peroxidase (TPO) antibodies and antinuclear antibodies systematically measured for all participants before pregnancy or at early pregnancy? Pre-pregnancy endometrial uterine NK cells will not be available in all participants.

8. Cost-effectiveness analysis. As both cost-effectiveness analysis (CEA) and cost-utility analysis (CUA) will be conducted. This section would be better renamed as “economic evaluations” to cover both analyses. It is confusing the author mentioned “methods such as cost-effectiveness ratios or cost-benefit analysis” at the end as “cost-benefit analysis” refers to a third type of economic evaluations which is not planned here. It would be good to clearly mention the time horizon and health outcomes for CEA and CUA, respectively.

9. Abstract: Please add the outcome rate in the comparator group in the abstract to make the sample size calculation reproduceable.

VERSION 1 - AUTHOR RESPONSE

Comments from reviewer 1 (dr. Xiaoxi Sun):

1. For patients with recurrent pregnancy loss, if the previous miscarriage was caused by fetal aneuploidy, would such patients be included?

Yes, women with potentially aneuploid losses are eligible for inclusion as fetal aneuploidy is more often not tested in case of pregnancy loss. We have included this in line 3-5 of the “participants and eligibility criteria” section.

2. Is biochemical pregnancy loss considered recurrent miscarriage?

Yes, biochemical losses are part of recurrent miscarriages as we follow the definition of the RPL ESHRE guideline.

3. A research status paragraph should be added in the paper.

We have now made it clear that the study is ongoing in the abstract and in the design and setting section.

Comments from reviewer 2 (dr. Rui Wang):

1. Timing of randomisation. According to page 7 Line 35-38, there are two possible timepoints for randomisation. “Patients may enrol in the study either during early pregnancy (<7 weeks of gestation) or before conception if they have an active desire to conceive. Both women who conceive spontaneously and those who conceive through artificial reproductive techniques are eligible for participation”. This will create a problem for the timing of randomisation, as those enrolled before conception would have been enrolled much earlier. In addition, for those enrolled before conception scheduled for ART, the time until conception will be uncertain as it may take multiple cycles before a conception occurs.

Thank you for your thoughtful comment. We would like to clarify that in this study, both inclusion and randomization may occur concurrently when women enroll after becoming pregnant. However, it is important to emphasize that women who enroll before pregnancy are only randomized to the intervention once pregnancy is confirmed. We have further clarified this distinction in the final paragraph of the "Participant and Eligibility" section to ensure greater clarity.

2. Duration of intervention and follow-up. Following #1, the duration of intervention and follow-up could be much longer for those who are enrolled before conception.

The treatment regimen is the same for all women, as they are only allocated to the treatment once pregnancy is confirmed. However, there is some variability in the timing of randomization, as women may be randomized between the time of pregnancy confirmation (as early as 4 weeks) and up to 6+6 weeks of gestation

3. Stratified randomisation. If the authors were to keep the inclusion of those enrolled before conception, a stratified randomisation considering the timing or randomisation is recommended and this should also be considered in the analysis.

None of the participants are randomized prior to conception. We hope this point has been clarified in our previous responses and in the "Participant and Eligibility" section. Stratified randomization was not feasible due to practical reasons, as the medication is now dispensed by a single pharmacy rather than multiple pharmacies corresponding to the participating centers.

4. Terminology. Miscarriage and pregnancy loss are interchangeably used across the protocol. It would be better to use "pregnancy loss" consistently across the protocol, in line with the ESHRE guideline on RPL.

We have made revisions throughout the entire manuscript in response to your comment. The term "pregnancy loss" has now been consistently used in place of "miscarriage."

5. Outcomes:

- There are some inconsistencies in the outcomes listed in the protocol and those in the trial registration, please make them consistent. We have checked our trial registration and have made adaptions for it to match the protocol.

- "gestational age at miscarriage (if applicable)" is defined as an outcome in the protocol. What about those with a live birth? How will this outcome be handled in the analysis? Gestational age at live birth is one of the outcomes of our study. We do not anticipate a difference between the prednisolone and placebo groups, as the treatment is administered early in pregnancy.

6. Statistical analysis:

- Any plans for per protocol or as-treated analysis? If so, how was "compliance" defined in this placebo-controlled trial? The primary analysis will follow the intention-to-treat principle; however, we also plan to conduct an as-treated analysis, as some participants may be unable to complete the full regimen if a miscarriage occurs before the final date of medication intake. A detailed statistical analysis plan (SAP) will be made publicly available in the coming months on our website (www.zorgevaluatienederland.nl/premi). Further details on the exact statistical methods will be outlined in the SAP.

- For secondary outcomes, please make it clear whether logistic regression adjusting for prognostic factors will be used for binary outcomes (most secondary outcomes) and whether estimate of the marginal treatment effect (absolute risk difference) will be presented.

We have followed your recommendation and added this to the secondary outcome section.

- Page 11, line 29-37. These refer to outcome definitions instead of statistical analysis and therefore should be merged to the "outcome measures" section.

We have merged the specified lines to the outcomes measures section.

7. Subgroup analysis: Are thyroid Peroxidase (TPO) antibodies and antinuclear antibodies systematically measured for all participants before pregnancy or at early pregnancy? Pre-pregnancy endometrial uterine NK cells will not be available in all participants.

You are correct that endometrial NK cells, TPO antibodies, and ANA are not part of the standard work-up for women with unexplained recurrent miscarriages, although they are occasionally measured. These outcomes are considered exploratory and are included as part of the study's broader objectives: they aim to lay the groundwork for future research with the aim of identifying specific biomarkers, which could potentially indicate where prednisolone may be more effective in these particular women. It is important to note that the study is not powered to analyze these subgroups. We have not made any specific changes to the manuscript in response to this comment, as it already clearly states that the effect is being "explored."

8. Cost-effectiveness analysis. As both cost-effectiveness analysis (CEA) and cost-utility analysis (CUA) will be conducted. This section would be better renamed as “economic evaluations” to cover both analyses. It is confusing the author mentioned “methods such as cost-effectiveness ratios or cost-benefit analysis” at the end as “cost-benefit analysis” refers to a third type of economic evaluations which is not planned here. It would be good to clearly mention the time horizon and health outcomes for CEA and CUA, respectively.

We have renamed the section previously titled "Economic Evaluations" and removed the cost-benefit analysis, as it was causing confusion. The time horizon for both analyses is currently difficult to predict. These analyses are planned as a separate publication and will be conducted in the year following the closure of the database.

9. Abstract: Please add the outcome rate in the comparator group in the abstract to make the sample size calculation reproduceable.

We have added the outcome rate in the comparator group in the abstract.

VERSION 2 - REVIEW

**Reviewer**	**2**
**Name**	**Wang, Rui**
**Affiliation**	**The University of Sydney, NHMRC Clinical Trials Centre**
**Date**	**24-May-2025**
**COI**	

I would like to thank the authors' for providing an excellent revision. I don't have further comments and look forward to seeing the protocol published and the results of the trial in the future.
